# Expression of Plasmodium vivax *crt-o* Is Related to Parasite Stage but Not *Ex Vivo* Chloroquine Susceptibility

**DOI:** 10.1128/AAC.02207-15

**Published:** 2015-12-31

**Authors:** Zuleima Pava, Irene Handayuni, Grennady Wirjanata, Sheren To, Leily Trianty, Rintis Noviyanti, Jeanne Rini Poespoprodjo, Sarah Auburn, Ric N. Price, Jutta Marfurt

**Affiliations:** a Global and Tropical Health Division, Menzies School of Health Research, Charles Darwin University, Darwin, Australia; b Eijkman Institute for Molecular Biology, Jakarta, Indonesia; c Papuan Health and Community Development Foundation (PHCDF), Timika, Papua, Indonesia; d Department of Paediatrics, Faculty of Medicine, Gadjah Mada University, Yogyakarta, Indonesia; e Centre for Tropical Medicine and Global Health, Nuffield Department of Clinical Medicine, University of Oxford, Oxford, United Kingdom

## Abstract

Chloroquine (CQ)-resistant Plasmodium vivax is present in most countries where P. vivax infection is endemic, but the underlying molecular mechanisms responsible remain unknown. Increased expression of P. vivax *crt-o* (*pvcrt-o*) has been correlated with *in vivo* CQ resistance in an area with low-grade resistance. We assessed *pvcrt-o* expression in isolates from Papua (Indonesia), where P. vivax is highly CQ resistant. *Ex vivo* drug susceptibilities to CQ, amodiaquine, piperaquine, mefloquine, and artesunate were determined using a modified schizont maturation assay. Expression levels of *pvcrt-o* were measured using a novel real-time quantitative reverse transcription-PCR method. Large variations in *pvcrt-o* expression were observed across the 51 isolates evaluated, with the fold change in expression level ranging from 0.01 to 59 relative to that seen with the P. vivax β-tubulin gene and from 0.01 to 24 relative to that seen with the P. vivax aldolase gene. Expression was significantly higher in isolates with the majority of parasites at the ring stage of development (median fold change, 1.7) compared to those at the trophozoite stage (median fold change, 0.5; *P* < 0.001). Twenty-nine isolates fulfilled the criteria for *ex vivo* drug susceptibility testing and showed high variability in CQ responses (median, 107.9 [range, 6.5 to 345.7] nM). After controlling for the parasite stage, we found that *pvcrt-o* expression levels did not correlate with the *ex vivo* response to CQ or with that to any of the other antimalarials tested. Our results highlight the importance of development-stage composition for measuring *pvcrt-o* expression and suggest that *pvcrt-o* transcription is not a primary determinant of *ex vivo* drug susceptibility. A comprehensive transcriptomic approach is warranted for an in-depth investigation of the role of gene expression levels and P. vivax drug resistance.

## INTRODUCTION

Chloroquine resistance (CQR) in Plasmodium vivax was first documented in 1989 in samples from the island of New Guinea (1, 2). Over the subsequent 25 years, evidence of reduced CQ susceptibility has been reported across most areas of the world where P. vivax infection is endemic (3), with significant consequences for the clinical manifestations of P. vivax infection and its control and management (4–6). Surveillance to identify areas where CQ-resistant P. vivax is emerging is critical but challenging, since the clinical response is confounded by high rates of relapse from dormant liver stages. Effective molecular markers would greatly improve surveillance strategies; however, the genetic basis of CQR in P. vivax remains unknown (7).

Molecular studies of CQ-resistant P. vivax have focused primarily on genes known to play a role in drug resistance in P. falciparum, particularly P. vivax *crt-o* (*pvcrt-o*) and *pvmdr1* (8, 9). Whereas several studies have shown no correlation between *pvcrt-o* single nucleotide polymorphisms (SNPs) and *in vivo* treatment outcome (10, 11), CQ transport and accumulation have been modified by transforming CQ-sensitive P. falciparum laboratory strains and Dictyostelium discoideum with *pvcrt-o* gene variants (12). In those studies, *pvcrt-o* wild-type transfected cells showed a 60% reduction in CQ accumulation compared to nontransfected control cells, but the 76T and 76L allelic variants did not increase further CQ tolerance. Furthermore, studies using the Saccharomyces cerevisiae expression system have demonstrated its ability to grow in the presence of CQ as a function of different P. falciparum chloroquine resistance transporter (PfCRT) and PvCRT allelic variants (13). Whereas yeast cells transfected with the CQ-resistant *pfcrt* haplotypes CIET (Dd2) and SNNT (7G8) showed CQ hypersensitivity compared to the CMNK wild type (HB3), CQ sensitivity was highest in *pvcrt-o* wild-type transfected cells.

Evidence for the role of *pvcrt-o* in modifying the clinical response to CQ was reported in a patient with severe malaria who had a 20-fold increase in *pvcrt-o* expression compared to three patients with nonsevere P. vivax infection (14). Furthermore, a Brazilian study of 44 clinical isolates demonstrated that *pvcrt-o* expression was elevated in patients with severe disease and poor response to CQ, leading the authors to hypothesize that altered expression levels of *pvcrt-o* could be mediating CQR in P. vivax (15).

The aim of the current study was to investigate the association between *pvcrt-o* expression level and *ex vivo* drug susceptibility in P. vivax field isolates from Papua (Indonesia), an area with high levels of CQ-resistant P. vivax (16, 17).

## MATERIALS AND METHODS

### Study site and subjects.

Clinical isolates were collected from patients presenting to the Rumah Sakit Mitra Masyarakat (RSMM) hospital with uncomplicated P. vivax monoinfection, with a peripheral parasitemia level of between 1,000 μl^−1^ and 80,000 μl^−1^ as determined by microscopic examination. After written informed consent was obtained, 5 ml of venous blood was collected by venipuncture and filtered using cellulose columns to deplete the host white blood cells (18). The postfiltration packed infected red blood cells (iRBC) were used for the *ex vivo* drug susceptibility assay and for RNA and DNA extraction.

### *Ex vivo* drug susceptibility.

The *ex vivo* susceptibility profiles of the antimalarials chloroquine (CQ), amodiaquine (AQ), mefloquine (MFQ), piperaquine (PIP), and artesunate (AS) were measured using a protocol modified from the World Health Organization (WHO) microtest as described previously (17, 19). *Ex vivo* drug susceptibility analyses were undertaken only in parasite isolates with more than 70% of parasites at the ring stage at the start of the assay as determined by microscopy (17, 20). In brief, 200 μl of a 2% hematocrit blood medium mixture (BMM), consisting of McCoy's 5A medium plus 20% matched human serum, was added to each well of predosed drug plates containing 11 serial concentrations (2-fold dilutions; maximum concentration shown in parentheses) of the antimalarials CQ (2,992 nM), piperaquine (PIP; 1029 nM), mefloquine (MFQ; 338 nM), amodiaquine (AQ; 158 nM), and artesunate (AS; 49 nM). A candle jar was used to mature the parasites at 37°C for 35 to 56 h. Incubation was stopped when >40% of ring-stage parasites had reached the mature schizont stage (i.e., >5 distinct nuclei per parasite) in the drug-free control well as determined by light microscopy. The dose-response data were analyzed using nonlinear regression analysis (WinNonLin 4.1; Pharsight Corporation, USA), and the value for the concentration inducing half-maximal inhibition of growth (IC_50_) was derived using an inhibitory sigmoid E_max_ model.

### RNA extraction and cDNA synthesis.

The iRBCs were mixed in a 1:10 volume ratio with TRIzol reagent (Invitrogen, Life Technologies, Scoresby, Australia) and stored at −80°C until use. Total RNA was extracted using a slightly modified version of the manufacturer's instructions in which the RNA precipitation step with isopropanol was extended overnight. RNA quality was assessed using the 260-nm/280-nm and 230-nm/280-nm ratios as determined on a NanoDrop UV spectrophotometer (Thermo Fisher Scientific, Scoresby, Australia), with values between 1.8 and 2 deemed acceptable. DNA digestion was performed by applying Ambion DNase treatment (Invitrogen), and the concentration of RNA was measured using the Quant-iT RNA assay (Invitrogen).

### Real-time quantitative reverse transcription-PCR (RT-qPCR).

cDNA synthesis was performed using Superscript III enzyme (Invitrogen) and oligo(dT)s (Invitrogen). A final quantity of 100 ng of total RNA was used in the reverse-transcription reaction following the manufacturer's instructions and cDNA was stored at −20°C until further processing.

A QuantiTect SYBR green PCR kit (Qiagen, Chadstone, Australia) was used to amplify cDNA. Primer-BLAST software and NetPrimer software were used to design primers spanning exon-exon junctions of the P. vivax β-tubulin gene and *pvcrt-o* (GenBank accession numbers XM_001615975 and XM_001613407, respectively); the primer pair described by Suwanarusk et al. (11) was used for the P. vivax aldolase gene (GenBank accession number AF247063) (Table 1). cDNA was amplified using a Rotor-gene 6000 series cycler (Corbett Life Science, Chadstone, Australia; software version 1.7) with the thermocycling conditions provided in the footnote of Table 1.

**TABLE 1:** Primer sequences and RT-qPCR cycling conditions^*a*^

Target	Primer sequence (5′→3′)	PCR product length (bp)
P. vivax β-tubulin gene	F: TTC CCCA ACA ACAC CAA GTCC	178
	R: TCC ATGC CCT CTCC TGT GTA	
P. vivax aldolase gene	F: GAC AGT G CCA CC AT CCT TACC	191
	R: CCT TCT CAA CAT TCT CCT TCT TTC C	
*pvcrt-o*	F: TTA TCTG CAT CCCG TCA TCC	159
	R: GTA TTTC GCT CTCA TTC TGTGC	

a PCR cycling conditions consisted of 95°C for 5 min followed by 40 cycles of denaturation at 95°C for 60 s, annealing at 60°C for 60 s, and extension at 72°C for 60 s.

### Assay validation. (i) Repeatability and reproducibility.

Samples were run in triplicate for each experiment and the final fold change (FC) of expression for a sample was derived from the results of at least two independent experiments. A CQ-sensitive isolate (CQ IC_50_ = 31.2 nM) with 82% of parasites at the ring stage of development and 18% of parasites at the trophozoite stage was used as the calibrator in all RT-qPCR runs. A threshold cycle (*C_T_*) value of 0.2 was applied to all the experiments. Intra-assay variation (repeatability) was evaluated by measuring the standard deviation (SD) of data from six replicates of the calibrator sample relative to the mean *C_T_* value. Interassay variation (reproducibility) of the FC values was calculated by means of the intraclass correlation coefficient, taking into account only isolates with results determined in at least three independent experiments.

### (ii) Sensitivity and specificity.

For simplicity, a theoretical 1:1 conversion of RNA to cDNA was assumed. The limit of detection (LOD) and the limit of quantification (LOQ) of the assays were established by evaluating triplicates of 10 2-fold serial dilutions of cDNA. Melt curve analyses were performed at the end of each run to confirm the specificities in each reaction by heating from 55 to 99°C with a ramping rate of 1°C/5 s. A random set of amplicons was sent for dideoxy sequencing to Macrogen Inc. (Geumcheon-gu, Seoul, Republic of Korea) to confirm correct target gene sequence amplification.

### (iii) Efficiency tests and internal control gene validation.

Standard curve analyses for each primer pair were performed and the corresponding efficiencies calculated using the formula *E* = 10^(−1/slope)^ (21), where *E* represents efficiency. Briefly, triplicates of 10 2-fold serial dilutions, starting from 100 ng, were evaluated in three independent experiments, using a different CQ-sensitive sample each time. The mean value of the efficiencies calculated in the three experiments was used to estimate the *pvcrt-o* expression levels relative to the levels seen with both control genes of each sample using the Pfaffl equation (22) and was compared with those calculated using the conventional 2^−ΔΔ*CT*^ formula (23), which assumes a PCR efficiency of 100%.

### Species determination assays and genotyping to identify polyclonal infections.

Genomic DNA (gDNA) was extracted from approximately 100 μl iRBC aliquots using a QIAamp DNA minikit (Qiagen). Plasmodium species confirmation was undertaken using a gDNA template and a modified version of the protocol of Padley et al. as described elsewhere (24, 25). Infection complexity was determined using data generated at three short tandem repeat (STR) markers: *Pv*3.27, MS16, and *msp1*F3. PCR amplification was undertaken on a gDNA template using the primer sequences, dye labels, PCR master mixes, and cycling conditions defined by Koepfli et al. (26). The fluorochrome-labeled PCR products were sized by denaturing capillary electrophoresis with LIZ-600 (Applied Biosystems) internal size standards using a service provider (Macrogen Inc.). The resulting electropherogram traces were analyzed using GeneMapper software (version 4.0; Applied Biosystems, Chadstone, Australia).To reduce potential artifacts arising from background noise, peaks below a cutoff of 100 relative fluorescent intensity units and peaks with less than 33% of the intensity of the highest peak were excluded. Infections with one or more loci displaying multiple alleles were defined as polyclonal; the remaining infections were defined as monoclonal.

### Data analysis.

Statistical analysis was undertaken using SPSS software (version 22; SPSS Inc., Chicago, IL, USA). Spearman's rank correlation and the Kruskal-Wallis test were used for nonparametric analysis. Agreement of the FC expression estimates between the two housekeeping genes was calculated by paired *t* test on log-transformed data. For categorical variables, percentages and corresponding 95% confidence intervals (CI) were calculated by assuming a binomial distribution.

### Ethics.

Ethical approval for this study has been obtained from the Eijkman Institute Research Ethics Commission, Eijkman Institute for Molecular Biology, Jakarta, Indonesia, and the Human Research Ethics Committee of the Northern Territory (NT) Department of Health & Families and Menzies School of Health Research, Darwin, Australia.

## RESULTS

### P. vivax field isolates and *ex vivo* drug susceptibility.

A total of 51 venous samples were collected, and the corresponding P. vivax field isolates were used for *ex vivo* drug susceptibility testing and molecular analysis. Baseline characteristics of these isolates are summarized in Table 2.

**TABLE 2:** Baseline characteristics of P. vivax isolates

Characteristic	No. of P. vivax isolates	Results
Parasitemia (no. of asexual parasites/μl)	51	Geometric mean, 7,561 (95% CI, 4,944–10,179)
Proportion of parasites at ring stage	29^*a*^	Median, 94% (range, 78%–100%)
Delay from venipuncture to processing	29	Median, 120 min (range, 50–225 min)
Duration of the drug assay	29	Median, 46 h (range, 25–54 h)
Schizont count at assay harvest	29	Mean, 40 (95% CI, 38.0–41.5)

a Number of isolates with ≥70% ring-stage parasites at the start of the *ex vivo* drug susceptibility assay.

Monospecies P. vivax infection was confirmed by PCR in all samples. Forty-three (84%) isolates could be genotyped successfully at all three STR loci, with 23 (53%) polyclonal infections identified. The remaining 20 (47%) isolates, including the CQ-sensitive calibrator, were from monoclonal infections. The allele frequencies for each marker are illustrated in Fig. S1 in the supplemental material.

### Assay validation.

The mean assay efficiencies were 1.93 (95% CI, 1.7 to 2.1) for *pvcrt-o*, 1.96 (95% CI, 1.8 to 2.1) for the P. vivax β-tubulin gene, and 1.94 (95% CI, 1.8 to 2.1) for the P. vivax aldolase gene. Sequencing of the amplicons confirmed the identity of each gene target and melt curve analyses produced single peaks for each gene. Whereas the LOD and LOQ of the P. vivax β-tubulin gene and the P. vivax aldolase gene were ≤0.2 ng cDNA, the lowest cDNA concentration tested in this study (mean *C_T_* value for the P. vivax β-tubulin gene of 28.2 [95% CI, 28.2 to 28.2] and mean *C_T_* value for the P. vivax aldolase gene of 21.9 [95% CI, 21.9 to 22.0], respectively), the LOD of *pvcrt-o* was 6.2 ng (mean *C_T_* value for *pvcrt-o*, 36.2 [95% CI, 26.3 to 46.2]) and the LOQ was 12.5 ng (mean *C_T_* value for *pvcrt-o*, 30.7 [95% CI, 29.7 to 31.8]). Overall, the P. vivax aldolase gene assay exhibited lower *C_T_* values than the P. vivax β-tubulin gene and *pvcrt-o* assays, with the *C_T_* values being similar in the latter two assays.

The mean *C_T_* values for the control sample across 14 experiments were 25.4 (95% CI, 25.1 to 25.7) for *pvcrt-o*, 23.9 (95% CI, 23.6 to 24.2) for the P. vivax β-tubulin gene, and 20.7 (95% CI, 20.4 to 21.1) for the P. vivax aldolase gene. *pvcrt-o* expression could be derived in 102 (81.6%) of the 125 assays conducted. The intraclass correlation coefficient (ICC) of the *pvcrt-o* FC of expression was 0.969 (95% CI, 0.798 to 0.990) relative to P. vivax β-tubulin gene expression and was 0.922 (95% CI, 0.843 to 0.962) relative to P. vivax aldolase gene expression. The *C_T_* values for the P. vivax β-tubulin gene and the P. vivax aldolase gene were highly correlated (*r* = 0.854, *P* < 0.001) (see Fig. S2A in the supplemental material). For all three genes, *C_T_* values were negatively correlated with the baseline parasitemia (*r* = −0.316 and *P* = 0.024 for the P. vivax β-tubulin gene, *r* = −0.333 and *P* = 0.017 for the P. vivax aldolase gene, and *r* = −0.433 and *P* = 0.002 for *pvcrt-o*) (see Fig. S2D to F).

**FIG 1:**
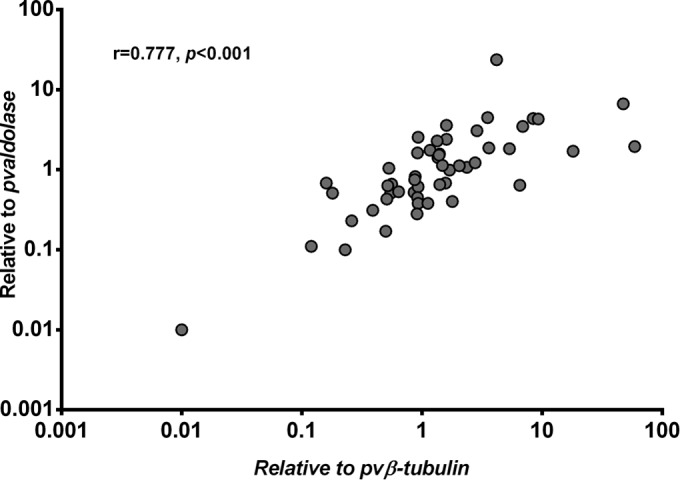
High correlation between *pvcrt-o* expression estimates derived with the P. vivax β-tubulin gene (*pvβ-tubulin*) and the P. vivax aldolase gene (*pvaldolase*). r, Pearson correlation coefficient.

The geometric mean FC of *pvcrt-o* expression was 1.3 (95% CI, −1.6 to 4.2; range, 0.01 to 59.0) relative to the expression of the P. vivax β-tubulin gene and 0.9 (95% CI, −0.05 to 1.8; range, 0.01 to 23.5) relative to the expression of the P. vivax aldolase gene. The two *pvcrt-o* FC estimates were highly correlated (*r* = 0.777, *P* < 0.001) (Fig. 1), although the FC values relative to the P. vivax β-tubulin gene were significantly higher than those for the P. vivax aldolase gene (median difference, 0.11; interquartile range, −0.172 to 0.932; *P* = 0.024).

### Expression levels of *pvcrt-o*, the P. vivax β-tubulin gene, and the P. vivax aldolase gene as a function of parasite stage.

**FIG 2:**
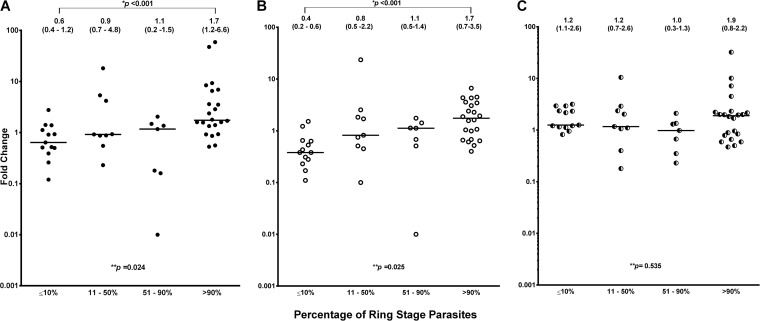
Expression levels of *pvcrt-o*, the P. vivax β-tubulin gene, and the P. vivax aldolase gene as a function of development stage. Data represent median (interquartile range) fold expression changes of *pvcrt-o* relative to the P. vivax β-tubulin gene (A), *pvcrt-o* relative to the P. vivax aldolase gene (B), and the P. vivax aldolase gene relative to the P. vivax β-tubulin gene (C) in isolates according to the proportion of parasites at the ring stage of development (**, Kruskal-Wallis test; *, Mann-Whitney U test). The horizontal lines represent the median fold change of gene expression.

There was a significant correlation between the FC of *pvcrt-o* expression and the proportion of ring-stage parasites (Spearman rank correlation coefficient [*r*_s_] = 0.474 [*P* < 0.001] relative to P. vivax β-tubulin gene expression and *r*_s_ = 0.516 [*P* < 0.001] relative to P. vivax aldolase gene expression). Comparing the *pvcrt-o* expression levels of isolates with a majority (>90%) of ring-stage parasites and isolates with a majority (>90%) of trophozoite-stage parasites, the mean expression levels were significantly higher in the group with a majority of ring-stage parasites (Student's *t* test statistic *t* = −2.16 [*P* = 0.038] relative to P. vivax β-tubulin gene expression; *t* = −2.64 [*P* = 0.012] relative to P. vivax aldolase gene expression). After classifying the 51 isolates according to the proportion of ring-stage parasites into groups with ≤10% (*n* = 13), 11% to 50% (*n* = 9), 51% to 90% (*n* = 7), and >90% (*n* = 22), a significant trend for increasing *pvcrt-o* expression levels with increasing proportion of ring-stage parasites was found, with the median FC of expression being 0.6 (interquartile range: 0.4 to 11.2) relative to P. vivax β-tubulin gene expression (Fig. 2A) and 0.4 (0.2 to 0.6) relative to P. vivax aldolase gene expression (Fig. 2B) in the group with ≤10% parasites at the ring stage of development (rings), compared to 1.7 (1.2 to 6.6) and 1.7 (0.7 to 3.5), respectively, in the group with >90% rings. Overall, there was a 3-fold difference between these two groups in median *pvcrt-o* expression levels.

In contrast, the relative levels of expression of the two housekeeping genes did not vary with parasite stage (Fig. 2C); neither did *pvcrt-o* expression differ significantly with infection complexity, the delay between venipuncture and sample processing, the duration of the drug assay, or the final schizont count after *ex vivo* culture.

### *pvcrt-o* expression and *ex vivo* drug susceptibility.

**FIG 3:**
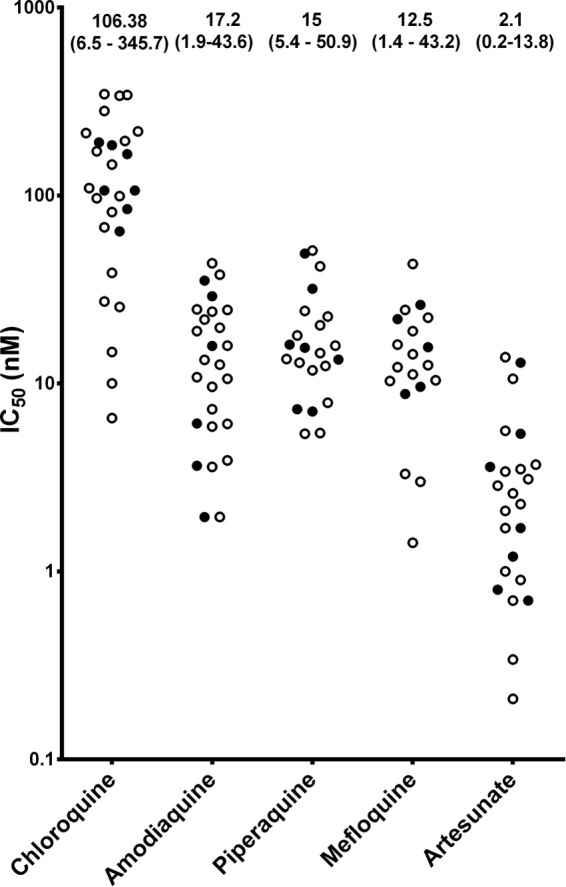
IC_50_s for each antimalarial drug with high and low expression of *pvcrt-o* relative to the P. vivax β-tubulin gene. Data represent the median (range) IC_50_s for each antimalarial. Results are shown for isolates with high-level (≥3.5-fold change; closed circles) or low-level (<3.5-fold change; open circles) expression of *pvcrt-o* relative to the P. vivax β-tubulin gene in P. vivax isolates with ≥70% rings at the start of the *ex vivo* drug susceptibility assay (*n* = 29).

**FIG 4:**
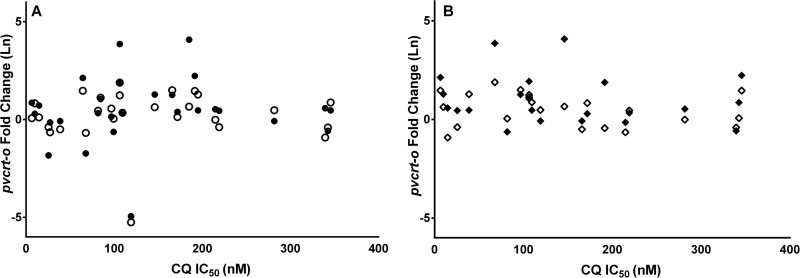
Relationship between *pvcrt-o* expression level and *ex vivo* drug susceptibility to CQ. (A) *pvcrt-o* expression relative to P. vivax β-tubulin gene expression (*r* = 0.106; *P* = 0.593; closed circles) and relative to P. vivax aldolase gene expression (*r* = 0.039; *P* = 0.843; open circles) versus *ex vivo* drug susceptibility to CQ in isolates with ≥70% ring-stage parasites at the start of the drug assay (*n* = 28). (B) *pvcrt-o* expression relative to P. vivax β-tubulin gene expression (*r* = 0.071; *P* = 0.760; closed diamond) and relative to P. vivax aldolase gene expression (*r* = 0.042; *P* = 0.856; open diamond) versus *ex vivo* drug susceptibility to CQ in isolates with ≥90% ring-stage parasites at the start of the drug assay (*n* = 21).

In total, 29 isolates fulfilled the criteria for *ex vivo* drug susceptibility testing, and IC_50_s could be derived in 28 isolates for CQ (median IC_50_, 107.9 [range, 6.5 to 345.7] nM), 23 for piperaquine (15.5 [5.4 to 50.9] nM), 20 for mefloquine (13.4 [1.4 to 43.2] nM), 27 for amodiaquine (18.4 [1.9 to 43.6] nM), and 25 for artesunate (2.6 [0.2 to 13.8] nM) (Fig. 3). There was no correlation between *pvcrt-o* expression and CQ IC_50_ relative to the expression of the P. vivax β-tubulin gene (*r* = 0.106, *P* = 0.593) or the P. vivax aldolase gene (*r* = 0.039, *P* = 0.843) (Fig. 4A), and this remained the case after applying a more stringent criterion in the 22 isolates with greater than 90% ring-stage parasites at the start of the assay (Fig. 4B) or in the 10 monoclonal infections. No correlation was observed between *pvcrt-o* expression and *ex vivo* drug susceptibility for any of the other drugs tested (see Fig. S3 in the supplemental material).

## DISCUSSION

*pfcrt* is a key determinant of CQR in P. falciparum (27), but the orthologous gene in P. vivax, *pvcrt-o*, remains poorly characterized and the evidence for its role in CQR is contentious. The current study evaluated for the first time the association between *pvcrt-o* expression and stage composition and the correlation between *pvcrt-o* expression and CQ *ex vivo* susceptibility in clinical isolates from a region where high-grade CQ-resistant P. vivax strains are endemic. Gene expression was assessed using a novel, reliable, and reproducible RT-qPCR assay, taking into account potential confounding factors such as parasitemia, stage composition, and complexity of infection. Large variations in *pvcrt-o* expression were observed across the 51 isolates evaluated, with the fold change (FC) expression ranging from 0.01 to 59 relative to the P. vivax β-tubulin gene and from 0.01 to 24 relative to the P. vivax aldolase gene.

The highly variable expression levels of *pvcrt-o* observed in our study most likely reflect differences in parasite stage composition in the isolates, with 3-fold-greater expression in isolates with a majority of ring-stage parasites than in those with a majority of trophozoite-stage parasites. However, the relative levels of expression of the housekeeping genes did not differ significantly with parasite stage, suggesting that the observations for *pvcrt-o* were determined predominantly by variations in *pvcrt-o* expression rather than by variations in expression of either of the housekeeping genes.

The degree of variation in expression was comparable to that observed by Melo et al. in clinical P. vivax isolates from the Brazilian Amazon, with FC levels ranging from 0.3 to 189 relative to the expression of the P. vivax β-tubulin gene (15) and to that reported for the expression of *pfcrt* in clinical P. falciparum isolates from Cambodia relative to the *ssu* gene (82.1; range, 1 to 5,713) and the *maebl* gene (194; range, 1 to 6,039) (28). Our results are also in concordance with those reported in studies of synchronized P. falciparum cultures, in which high expression of *pfcrt* was observed in ring-stage parasites, decreasing gradually throughout the second half of the life cycle (28, 29). However, while the expression levels of P. vivax genes are known to vary markedly during the parasite life cycle (30, 31), data on *pvcrt-o* expression throughout the P. vivax life cycle are limited (32).

In contrast to the previous study by Melo and colleagues (15), the analysis of the Papuan isolates showed no association between *pvcrt-o* expression and *ex vivo* susceptibility to CQ or any of the other standard antimalarials tested. Key differences between the two studies include different transmission intensities, differences in the drug susceptibility phenotypes and profiles, and different levels of parasite synchronicity. In the current study, the relationship between *pvcrt-o* expression levels and *ex vivo* drug response was assessed in highly synchronous isolates with proportions of rings between 78% and 100%, whereas in the Brazilian study, the proportion of ring-stage parasites in the isolates ranged from 29% to 88%. Furthermore, drug susceptibility was at a relatively low level in the Amazon, with treatment failure occurring in less than 12% of patients treated with CQ, whereas in Papua in 2007, 15% of patients had early treatment failure following CQ and more than 65% failed treatment by day 28 (16). In this region, the parasite stage composition along with the duration of the assay is an important determinant of *ex vivo* CQ susceptibility (17, 20). Using a standardized assay format, isolates from Papua, where the level of CQR is high, were up to 4-fold less susceptible to CQ than isolates from Thailand, where CQR is much less prevalent (11). Furthermore, the derived IC_50_s for CQ were up to 50-fold higher in P. vivax isolates predominantly at the trophozoite stage compared to those at the ring stage (33). Although a similar relationship has also been observed in P. malariae assays, the degree of stage specificity appears to be significantly less than that observed in P. falciparum (7). There is evidence that parasite staging also affects the clinical response. In Thailand, patients with schizonts present on the initial blood films were five times more likely to remain parasitemic at hour 24 compared to those with parasites predominantly at the early and late trophozoite stages (34). Since *ex vivo* and *in vivo* drug responses may be associated with parasite stage, any correlation of parasite molecular markers and drug resistance phenotypes needs to control for potential confounding by stage composition.

Polyclonal infections can also confound the interpretation of *pvcrt-o* expression estimates, depending upon the relative proportions of the strains present. One limitation of the study was the high (56%) proportion of polyclonal P. vivax infections. However, after restricting data analysis to monoclonal infections, there was still no correlation between *pvcrt-o* expression levels and drug susceptibility or, indeed, a trend to significance.

Quantification of gene expression by RT-qPCR is reliant upon the choice of suitable housekeeping genes. The P. vivax β-tubulin gene and P. vivax aldolase gene, the two housekeeping genes used in the current study, were selected on the basis of available data in PlasmoDB, showing relatively stable expression of the P. vivax aldolase gene between hours 0 to 20 and of the P. vivax β-tubulin gene between hours 23 to 40 of the 48-hour life cycle (32). The statistically significant (albeit small) difference between the *pvcrt-o* expression estimates obtained using these two control genes highlights the need to test and evaluate multiple housekeeping genes in the early phase of development of assays of relative quantification in Plasmodium.

Two studies have shown evidence for the involvement of the PvCRT protein in CQ transportation and accumulation (12, 13). Functional studies of the PfCRT and PvCRT proteins in S. cerevisiae yeast expression models by Baro and colleagues showed that wild-type PvCRT is already as efficient for CQ efflux as PfCRT variants associated with a CQ resistance phenotype. As a consequence, additional mutations to increase the efficiency of transportation of CQ across the digestive vacuole membrane, in order to confer a CQ resistance phenotype, do not seem to be necessary. Together with the observations from the current study, there is growing evidence that *pvcrt-o* may have a role in mediating CQR mechanisms in P. vivax that is different from that of *pfcrt* in P. falciparum.

In conclusion, we found no evidence that *pvcrt-o* expression modified *ex vivo* susceptibility to CQ in isolates from an area endemic for high-grade CQ-resistant P. vivax strains. Our results suggest that parasite staging is an important confounding factor that needs to be addressed in quantifying gene transcription levels in clinical Plasmodium field isolates. However, these observations do not rule out a possible role of *pvcrt-o* in a multigenic modulation of P. vivax CQR. Transcriptome analyses are needed to shed light on the precise role of *pvcrt-o*, and other loci, in conferring CQR in P. vivax.

## Supplementary Material

Supplemental material
