# Supplementary material for: Expression of Plasmodium vivax crt-o Is Related to Parasite Stage but Not Ex Vivo Chloroquine Susceptibility
Source: Antimicrob Agents Chemother. 2015 Dec 31;60(1):361–7. doi: 10.1128/AAC.02207-15 (PMC4704153; doi:10.1128/AAC.02207-15)
Supplement: Supplemental material [file supp_60_1_361__index.html]

Expression of Plasmodium vivax crt-o Is Related to Parasite Stage but Not Ex Vivo Chloroquine Susceptibility — Supplemental material 

# Expression of Plasmodium vivax*crt-o* Is Related to Parasite Stage but Not *Ex Vivo* Chloroquine Susceptibility

## Supplemental material

- Supplemental file 1 -

  Fig. S1-S3

  PDF, 396K
