# Supplementary material for: Expression of Plasmodium vivax crt-o Is Related to Parasite Stage but Not Ex Vivo Chloroquine Susceptibility
Source: Antimicrob Agents Chemother. 2015 Dec 31;60(1):361–7. doi: 10.1128/AAC.02207-15 (PMC4704153; doi:10.1128/AAC.02207-15)
Supplement: Supplemental material [file AAC.02207-15_zac001164744so1.pdf]

Supplemental Material

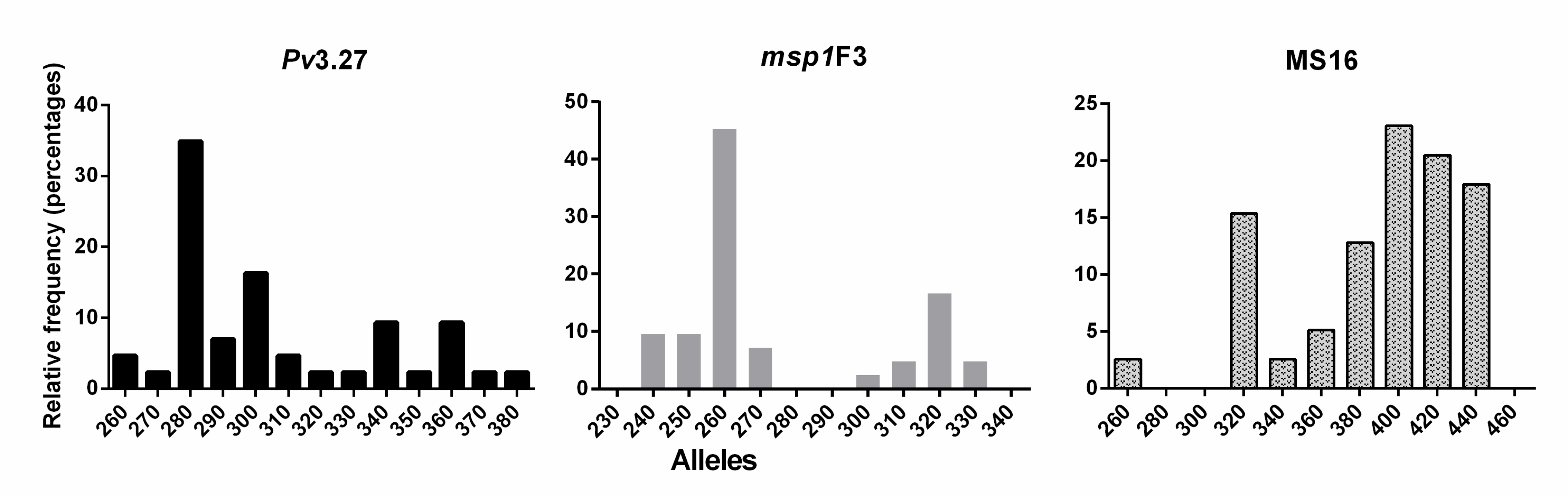

Figure S1. Allele frequency distributions of short tandem repeat (STR) markers *Pv*3.27, MS16 and *msp1*F3.

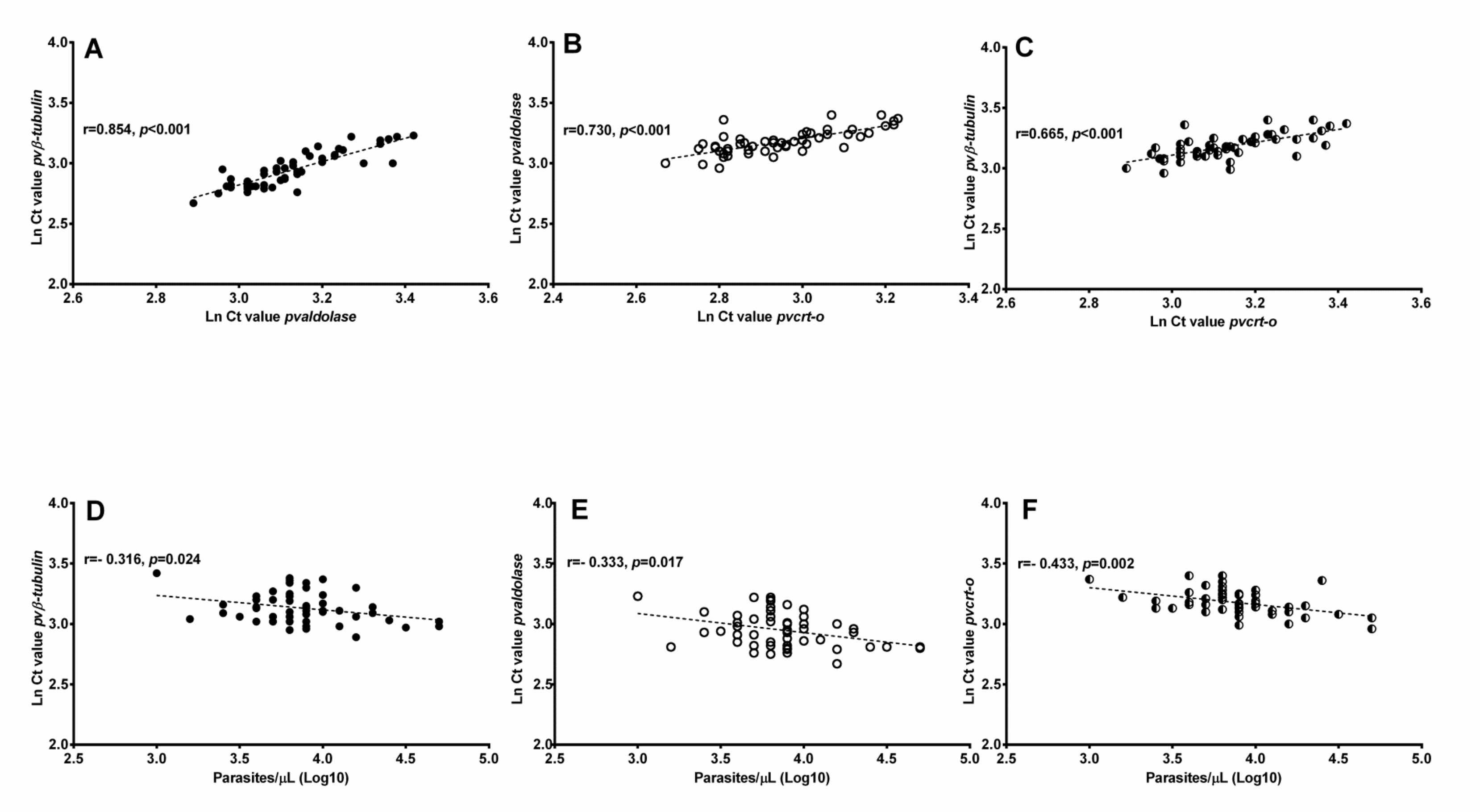

Figure S2. Positive correlation between log-transformed Ct values of *pvcrt-o*, *pvβ-tubulin*, and *pvaldolase* (A-C), and negative correlation between the log-transformed Ct values of *pvβ-tubulin* (D), *pvaldolase* (E), and *pvcrt-o* (F) and parasitaemia.

Footnote: r, Pearson correlation coefficient

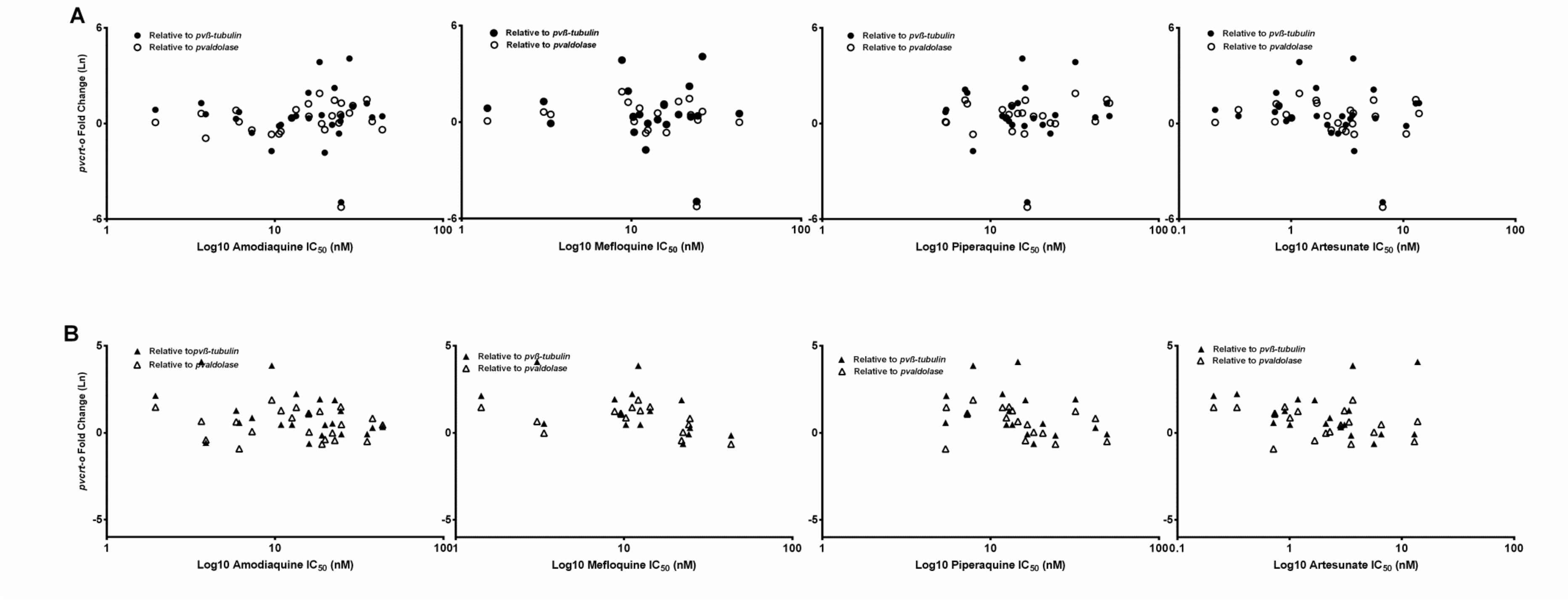

Figure S3: Relationship between *pvcrt-o* expression levels and *ex vivo* drug susceptibility to all antimalarials tested.

Footnote: Correlation of *ex vivo* response in *P. vivax* isolates with ≥70% ring stage parasites (A) to amodiaquine and *pvcrt-o* expression relative to *pvβ-tubulin* (r=0.120, *p*=0.954) and relative to *pvaldolase* (r=0.120, *p*=0.954), mefloquine and *pvcrt-o* expression relative to *pvβ-tubulin* (r=-0.089, *p*=0.710) and relative to *pvaldolase* (r=-0.170, *p*=0.474), piperaquine and *pvcrt-o* expression relative to *pvβ-tubulin* (r=-0.033, *p*=0.880) and relative to *pvaldolase* (r=-0.115, *p*=0.602), and artesunate and *pvcrt-o* expression relative to *pvβ-tubulin* (r=-0.182, *p*=0.385) and relative to

pvaldolase (r=-0.241, *p*=0.247). Correlation of *ex vivo* response in *P. vivax* isolates with >90% ring stage parasites (B) to amodiquine and *pvcrt-o* expression relative to *pvβ-tubulin* (r=0.199, *p*=0.401) and relative to *pvaldolase* (r=0.379, *p*=0.0994), mefloquine and *pvcrt-o* expression relative to *pvβ-tubulin* (r=0.116, *p*=0.681) and relative to *pvaldolase* (r=-0.053, *p*=0.850), piperaquine and *pvcrt-o* expression relative to *pvβ-tubulin* (r=-0.064, *p*=0.806) and relative to *pvaldolase* (r=-0.206, *p*=0.427), and artesunate and *pvcrt-o* expression relative to *pvβ-tubulin* (r=-0.072, *p*=0.763) and relative to *pvaldolase* (r=-0.145, *p*=0.541).
